# Carbon Dioxide and Water Electrolysis Using New Alkaline Stable Anion Membranes

**DOI:** 10.3389/fchem.2018.00263

**Published:** 2018-07-03

**Authors:** Jerry J. Kaczur, Hongzhou Yang, Zengcai Liu, Syed D. Sajjad, Richard I. Masel

**Affiliations:** Dioxide Materials Inc., Boca Raton, FL, United States

**Keywords:** anion exchange membranes, electrochemical, formic acid, carbon monoxide, CO_2_ utilization, alkaline water electrolysis

## Abstract

The recent development and market introduction of a new type of alkaline stable imidazole-based anion exchange membrane and related ionomers by Dioxide Materials is enabling the advancement of new and improved electrochemical processes which can operate at commercially viable operating voltages, current efficiencies, and current densities. These processes include the electrochemical conversion of CO_2_ to formic acid (HCOOH), CO_2_ to carbon monoxide (CO), and alkaline water electrolysis, generating hydrogen at high current densities at low voltages without the need for any precious metal electrocatalysts. The first process is the direct electrochemical generation of pure formic acid in a three-compartment cell configuration using the alkaline stable anion exchange membrane and a cation exchange membrane. The cell operates at a current density of 140 mA/cm^2^ at a cell voltage of 3.5 V. The power consumption for production of formic acid (FA) is about 4.3–4.7 kWh/kg of FA. The second process is the electrochemical conversion of CO_2_ to CO, a key focus product in the generation of renewable fuels and chemicals. The CO_2_ cell consists of a two-compartment design utilizing the alkaline stable anion exchange membrane to separate the anode and cathode compartments. A nanoparticle IrO_2_ catalyst on a GDE structure is used as the anode and a GDE utilizing a nanoparticle Ag/imidazolium-based ionomer catalyst combination is used as a cathode. The CO_2_ cell has been operated at current densities of 200 to 600 mA/cm^2^ at voltages of 3.0 to 3.2 respectively with CO_2_ to CO conversion selectivities of 95–99%. The third process is an alkaline water electrolysis cell process, where the alkaline stable anion exchange membrane allows stable cell operation in 1 M KOH electrolyte solutions at current densities of 1 A/cm^2^ at about 1.90 V. The cell has demonstrated operation for thousands of hours, showing a voltage increase in time of only 5 μV/h. The alkaline electrolysis technology does not require any precious metal catalysts as compared to polymer electrolyte membrane (PEM) design water electrolyzers. In this paper, we discuss the detailed technical aspects of these three technologies utilizing this unique anion exchange membrane.

## Introduction

Over the past decade, increasing interest has been directed in utilizing carbon dioxide in generating liquid fuels and chemicals as a means toward a sustainable, carbon-neutral based economy. The efficient generation of energy-dense carbon-based products from captured and anthropogenic CO_2_ using renewable energy sources such as solar energy, wind, nuclear, and hydroelectric provides the basis for sourcing sustainable chemical feedstocks that are not derived from fossil fuels (Halmann, 1993; Aresta and Dibenedetto, 2007; Aresta, 2010; Whipple and Kenis, 2010; Quadrelli et al., 2011; Sankaranarayanan and Srinivasan, 2012; Hu et al., 2013; Masel et al., 2014a, 2016a; Aresta et al., 2016).

The efficient generation of carbon monoxide (CO) from CO_2_ is one of the key focus areas in producing sustainable chemical feedstocks. It is an industrially valuable chemical that is typically produced from methane, producing various mixture ratios of CO and hydrogen, called syngas. Syngas is used in the manufacture of fuels, such as diesel and gasoline, as well as other chemicals via Fischer-Tropsch (F-T) chemistry using selected catalysts and operating conditions (Appel, 2013; Costentin et al., 2014; Masel et al., 2014a, 2016a; Liu et al, 2015).

The generation of renewable hydrogen is the other key renewable energy product focus area. Hydrogen is used in many industrial processes and a component of the “Hydrogen Economy” concept. It is a way of storing and generating renewable energy that can be used as a fuel for automobiles and other applications (Ursua et al., 2012; U.S. Department of Energy (DOE), 2018).

## New alkaline stable anion membranes

Dioxide Materials (DM) has developed a group of novel imidazole-functionalized membranes using a polystyrene-based backbone that are surprisingly stable in strong alkaline solutions (Masel et al., 2017a,b,c). These membranes are now available for researchers for developing other process applications. Details on these membranes are provided in the next sections.

### Sustainion® pre-polymer and anion membrane description

**Figure 1:**
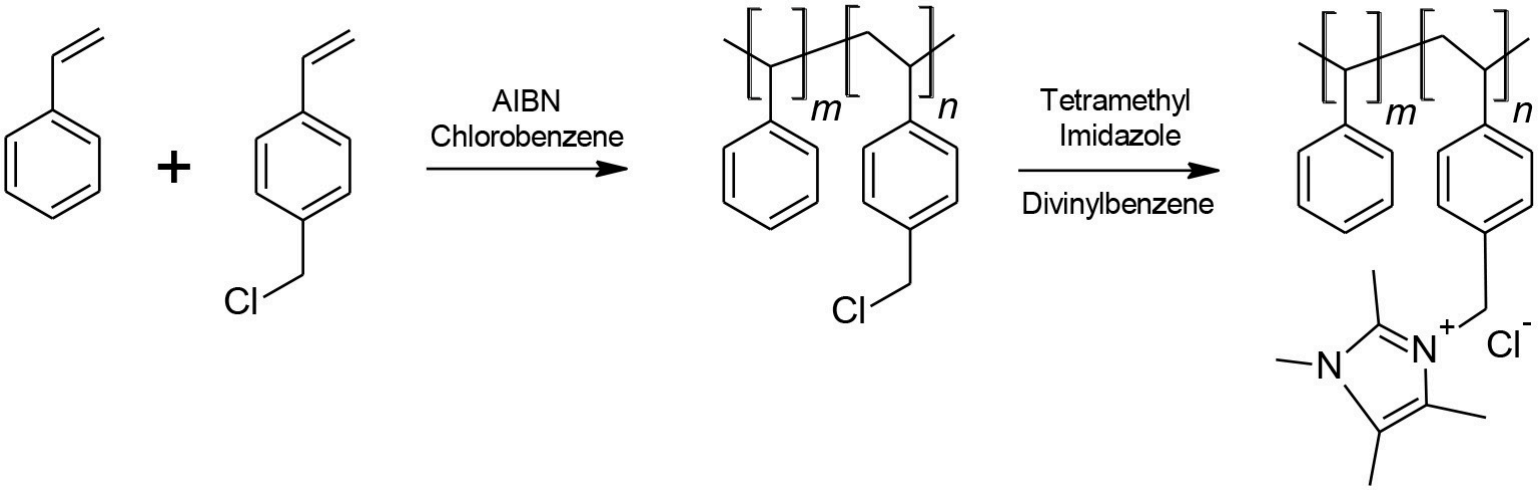
Synthetic route of the pre-polymer in fabricating Sustainion® anion exchange membranes.

The anion exchange Sustanion® membranes are based on a cheap and abundant, but more importantly, alkaline stable polystyrene backbone. The polymer synthesis and subsequent fabrication of the membrane have been described in detail in several past publications (Masel et al., 2016b, 2017d; Kutz et al., 2017; Liu et al., 2017a; Sajjad et al., 2017). The synthesis involves a two-step process (Figure 1) of copolymerization followed by subsequent functionalization. The membrane can be cast as a film or as a reinforced membrane using various reinforcement materials. The chloride form of the membrane is then typically converted to the hydroxide form using KOH.

Briefly, a copolymer of styrene and vinyl benzyl chloride is prepared by free radical addition polymerization. The copolymer product is then washed and precipitated in ethanol. Afterwards, it is filtered, dried and subsequently functionalized with 1,2,4,5-tetramethylimidazole in a Dowanol® PM (1-methoxy-2 propanol) solvent. Divinylbenzene is also added as a crosslinker to help improve membrane strength. The cast membrane is then activated for 8–16 h in a 1M potassium hydroxide solution for conversion to the hydroxide form.

### Membrane production scale-up

The synthetic route for the ionomer shown in Figure 1 has been ramped up to industrial sized reactor volumes and transferred to a commercial size pilot coater for membrane manufacturing. A membrane production run typically produces several hundred to a thousand feet of membrane having a width of about 24 cm. The final width and total membrane footage can be altered according to customer requirements.

### Anion membrane properties

The anion exchange membranes are typically supplied in a dry, chloride form supported on a polyethylene terephthalate (PET) liner. They consequently have to be converted to the hydroxide form by exposing them to a strong base. DM recommends soaking the membrane in a large bath of 1M KOH for 24–48 h in order to convert the membrane chloride form into the hydroxide form. This activation also helps in strengthening the membrane via crosslinking with KOH as identified in a recent nuclear magnetic spectroscopy (NMR) study (Pellerite et al., 2017). Furthermore, during this process the membrane swells and separates from the PET liner, which is discarded.

The membrane has an ion exchange capacity (IEC) of ~1.1 mmol/g calculated through standard back titration technique (Vengatesan et al., 2015). Even more impressive is the area specific resistance (ASR) of the membrane. Table 1 shows that the measured normalized resistance for Sustanion® 37–50 at the same alkaline conditions is more than an order lower than the other commercially available membranes. The anion exchange membrane has shown excellent performance ability in multiple cell runs of 1,000–3,000 h and more of run time in electrochemical cell testing in a wide pH range as exemplified in published experimental carbon dioxide and alkaline water electrolyzer data (Masel et al., 2016b; Kutz et al., 2017; Liu et al., 2017a,b; Sajjad et al., 2017). Some of the latest results are highlighted in later sections of this paper.

**Table 1:** Area specific resistance (ASR) measurements of Sustanion® 37-50 and other commercial ion exchange membranes in 1M KOH at 60°C.

**Membrane**	**ASR in 1 M KOH at 60°C (Ω-cm^2^)**	**Membrane Operational pH Range (Mfg. Spec Sheet)**
Sustainion® 37-50	0.045	2–14
Nafion® N115	0.52	0–13
Fumasep FAPQ-375	0.83	0–11
AMI-7001	2.0	0–10
PBI (polybenzimidazole)	8.3	2–10
Neosepta® ACN	>50	0–8

The Sustainion® anion exchange membranes undergo moderate swelling, about 5% in lateral directions and about 50% in thickness during conversion to the hydroxide form using 1M KOH. This membrane swelling or high-water uptake (~80%) explains the low area specific resistance (ASR) (i.e., high ionic conductivity) measurement values, as water absorption seems an important mechanism for hydroxide ion transport. Recognizing the need for more dimensionally stable and robust membranes, DM has now been also producing more robust reinforced versions of the Sustanion® anion exchange membranes, suitable for use in larger electrolyzers and more demanding electrochemical applications. The reinforcement support makes these membranes stronger in both the wet and dry states.

## Electrochemical conversion of CO_2_ to formic acid

Numerous researchers over the past 30 years have been examining the performance of various catalysts in the electrochemical reduction conversion of CO_2_ to formate/formic acid. Various papers have provided excellent summary reviews on previous experimental work (Jitaru, 2007; Rosen et al., 2011; Lu et al., 2014; Qiao et al., 2014; Pletcher, 2015; Du, 2017) and will not be further described here. More recently, a number of investigators have gone one step further, conducting and reporting studies providing performance data on complete electrochemical CO_2_ cell conversion performance in producing formate/formic acid (Mahmood et al., 1987; Li and Oloman, 2005, 2006, 2007; Oloman and Li, 2008; Whipple et al., 2010; Agarwal et al., 2011; Alvarez-Guerra et al., 2012, 2014; Kopljar et al., 2014; White et al., 2014; Du et al, 2016; Sen et al., 2016).

DM has been able to successfully produce high concentrations of pure formic acid directly in an electrochemical cell from the electrochemical reduction of CO_2_ (Kaczur et al., 2017; Yang et al., 2017a,b). This reduces the need for acid conversion of alkali metal formate salt-based products (e.g., potassium formate) which are typically produced using these alternative electrochemical cell and process configurations.

### Formic acid (FA) cell design

The DM electrochemical formic acid cell configuration is based on a three-compartment design consisting of an anode compartment, a center flow compartment containing a cation ion exchange media where the formic acid product is collected and removed from the cell, and a cathode compartment where the electrochemical reduction of CO_2_ to formate ions occurs (Kaczur et al., 2017; Yang et al., 2017a,b).

**Figure 2:**
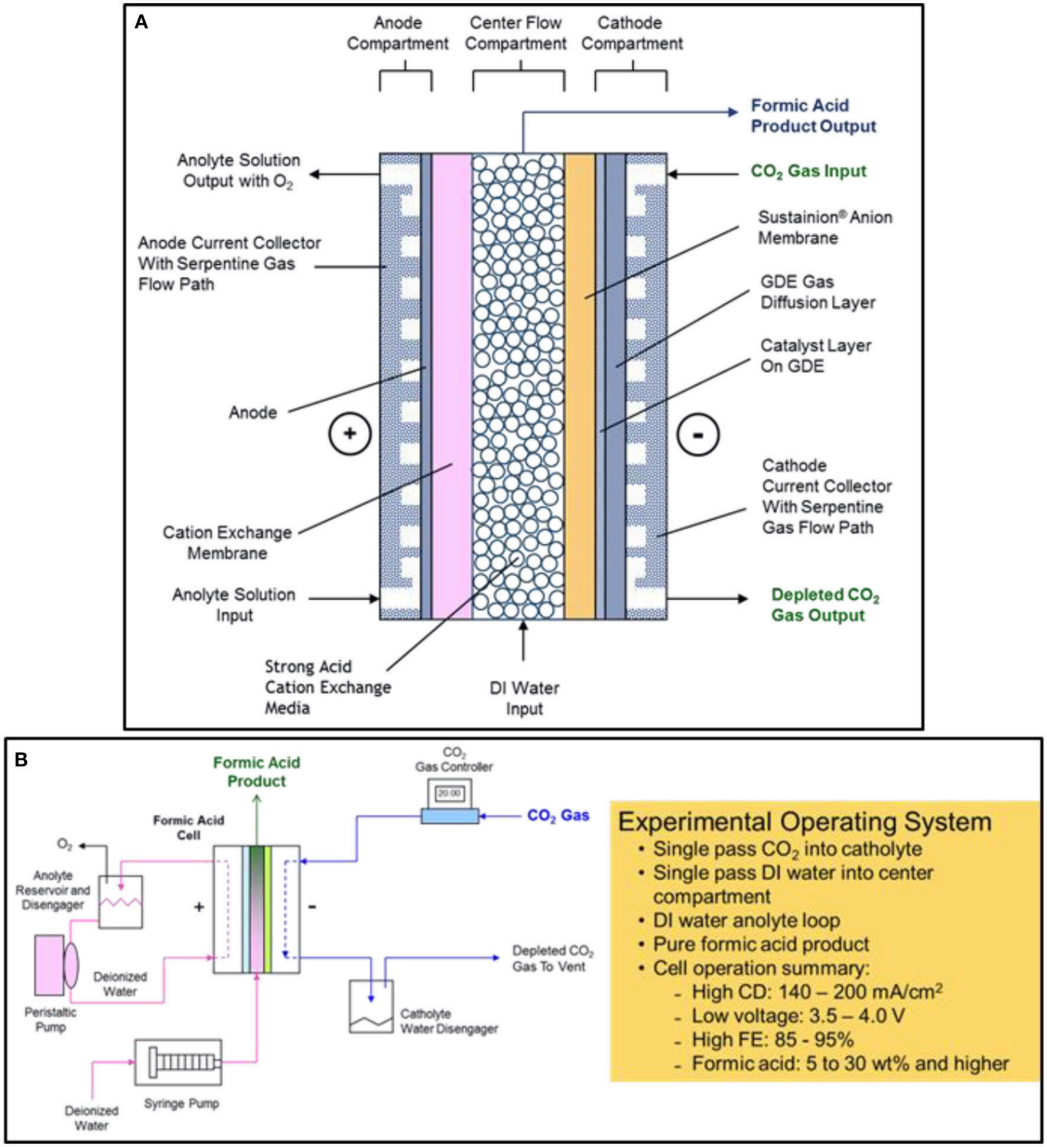
The electrochemical three compartment formic acid cell design configuration is shown in **(A)**. The experimental formic acid cell operating system and performance summary is shown in **(B)**.

The general electrochemical formic acid cell configuration is shown in Figure 2A. The anode compartment utilizes an MMO (mixed metal oxide) coated titanium anode where deionized water is electrolyzed producing oxygen and hydrogen ions (H^+^). A cation ion exchange membrane adjoining the anode compartment is used to block the transport of formate anions to the anode, where it would be oxidized to CO_2_. The formed hydrogen ions pass through the cation exchange membrane into the center flow compartment. The preferred design cation membrane is a perfluorinated sulfonic acid type membrane which is both oxidation stable and efficient in blocking formate anion transport. The anolyte uses a deionized water anolyte since the anode-membrane has a zero-gap contact arrangement.

In the cathode compartment, CO_2_ is efficiently reduced at low overpotentials to formate using a tin nanoparticle and imidazole-based ionomer catalyst coating combination in a carbon GDE (gas diffusion electrode) structure. The formate ions produced, as well as hydroxide and bicarbonate/carbonate ions that are formed, then pass through the adjoining Sustainion® anion exchange membrane into the center flow compartment.

In the center flow compartment, a cation ion exchange media is used to provide the solution conductivity needed for the formic acid product that is being formed in the compartment. Formate ions formed in the cathode compartment pass through the adjoining Sustainion® anion membrane and combine with the hydrogen ions transporting through the cation exchange membrane from the anode compartment to form a pure formic acid product. Deionized water metered into the center flow compartment is used to collect and remove the formic acid product from the compartment. The formic acid concentration depends on the input water flowrate into the center flow compartment and the operating cell current, forming a formic acid end product at concentrations that can range from 2 to 30 wt% and at high Faradaic efficiencies of 85 to 95%. Any hydroxide ions entering the center flow compartment react with hydrogen ions to form water, and any bicarbonate/carbonate ion transport will react with the hydrogen ions to form CO_2_, exiting with the formic acid product as gaseous CO_2_. The cation ion exchange media used in the design, Amberlite® IR120 hydrogen form cation ion exchange resin beads, provided improved ionic conductivity in the center flow compartment since formic acid solutions have a significantly lower conductivity. The cation exchange media provided a lower operating cell voltage (Yang et al., 2017a).

### Formic acid cell experimental operating system

Figure 2B shows a schematic of the experimental formic acid cell operating system as well as a brief summary of the cell operating performance. The cell produced formic acid concentrations of between 5 to 30% depending on the single pass flow rate of DI water feed into the center compartment. The FA cell operating current density range is from 140 to 200 mA cm^−2^ at corresponding cell voltages of 3.5 and 4.0 volts operating at room temperature. The Faradaic cell efficiency ranged from 80 to 95% when utilizing a Nafion® 324 cation exchange membrane and a Sustainion® 37-50 anion exchange membrane in the cell configuration (Yang et al., 2017a).

### Formic acid cell chemistry

**Figure 3:**
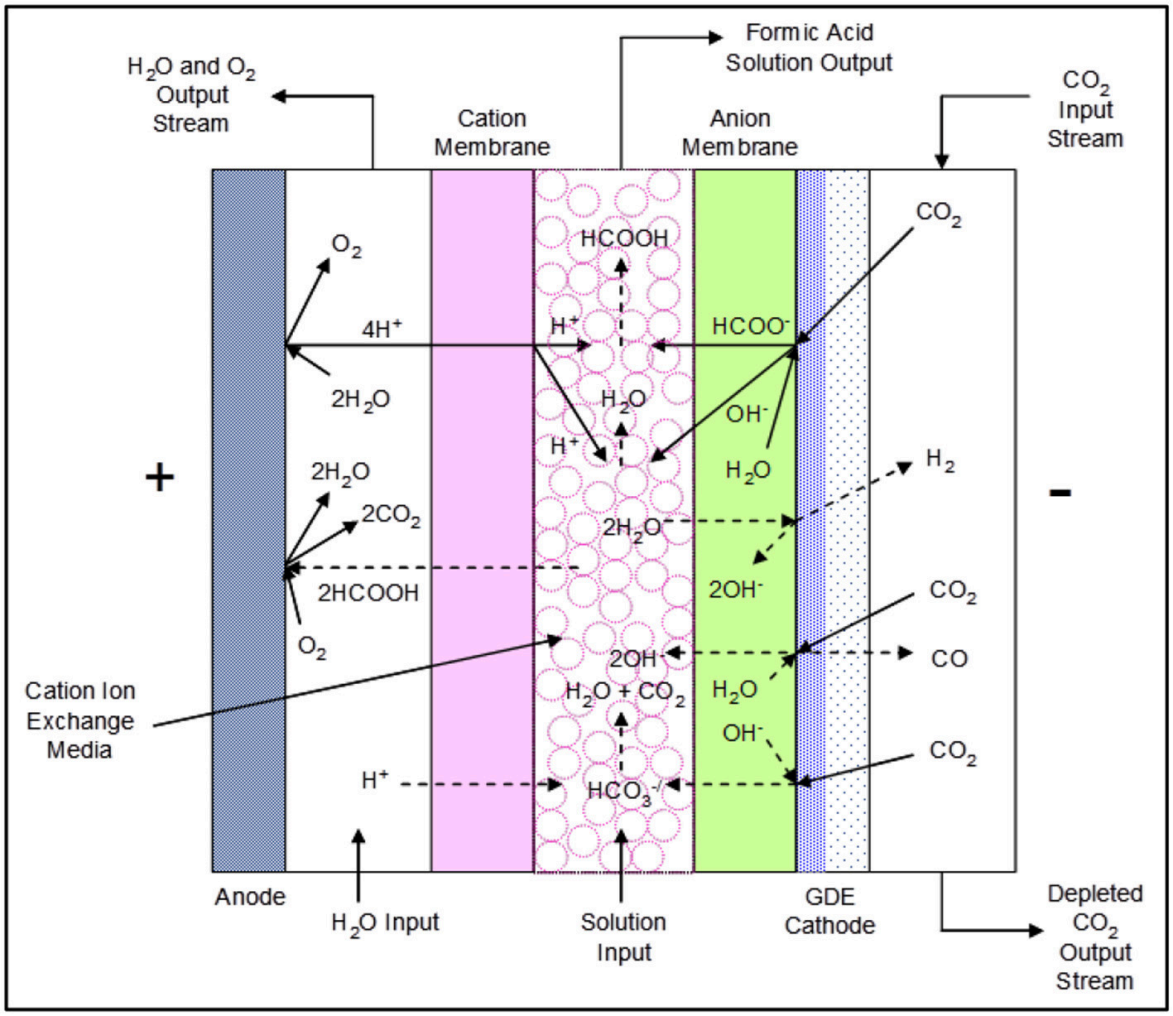
The DM 3-compartment formic acid cell configuration showing proposed reactions and ion transport.

The formic acid (FA) cell has a complex set of reactions that occur at the anode, the GDE cathode, and the center flow compartment that is bounded by a cation exchange membrane on the anode side and an anion exchange membrane on the cathode side. Figure 3 shows the proposed main and secondary reactions as well as ion transport that may occur during cell operation.

#### Formic acid cell main reactions

The electrochemical reduction of CO_2_ occurs in the presence of water at the cathode, forming formate (HCOO^−^) and hydroxide (OH^−^) ions:

(1) $${\text{CO}}_{\text{2}}+{\text{H}}_{2}\text{O}+{\text{2e}}^{-}\to {\text{HCOO}}^{-}+{\text{OH}}^{-}$$

Simultaneously, the oxidation of water occurs at the anode, forming oxygen gas and hydrogen ions (H^+^). Hydrogen ions are actually hydronium or oxonium cations (H_3_O^+^) in aqueous solutions, and in this paper we are using H^+^ as the substitute for hydronium ions.

(2) $${\text{2H}}_{\text{2}}\text{O}\to {\text{4H}}^{+}+{\text{4e}}^{-}+{\text{O}}_{2}$$

Both formate ions and hydroxide ions migrate through the anion exchange membrane into the center flow compartment, where they combine with hydrogen ions produced in the anode compartment passing through the cation membrane to yield water and formic acid as follows:

(3) $${\text{H}}^{+}+{\text{OH}}^{-}\to {\text{H}}_{2}\text{O}$$

(4) $${\text{H}}^{+}+{\text{HCOO}}^{-}\to \text{HCOOH}$$

#### Formic acid cell side reactions

Side reactions that can occur at the cathode and catholyte are as follows:

(5) $${\text{CO}}_{\text{2}}+{\text{H}}_{2}\text{O}+{\text{2e}}^{-}\to \text{CO}+{\text{2OH}}^{-}$$

(6) $${\text{2H}}_{\text{2}}\text{O}+{\text{2e}}^{-}\to {\text{H}}_{2}+{\text{2OH}}^{-}$$

(7) $${\text{CO}}_{\text{2}}+{\text{OH}}^{-}\to {\text{HCO}}_{\text{3}}^{-}$$

Bicarbonate (${\text{HCO}}_{3}^{-}$) anions formed at the cathode layer migrate through the anion exchange membrane into the center flow compartment, reacting with hydrogen ions entering the center flow compartment from the anode compartment through the adjoining cation membrane to produce CO_2_:

(8) $${\text{H}}^{+}+{\text{HCO}}_{\text{3}}^{-}\to {\text{CO}}_{\text{2}}+{\text{H}}_{2}\text{O}$$

Formic acid product will be lost if it transports through the cation membrane into the anolyte compartment, being subsequently oxidized at the anode to CO_2_:

(9) $$\text{HCOOH}\to {\text{CO}}_{\text{2}}+{\text{2H}}^{+}+{\text{2e}}^{-}$$

### Formic acid cell performance

**Figure 4:**
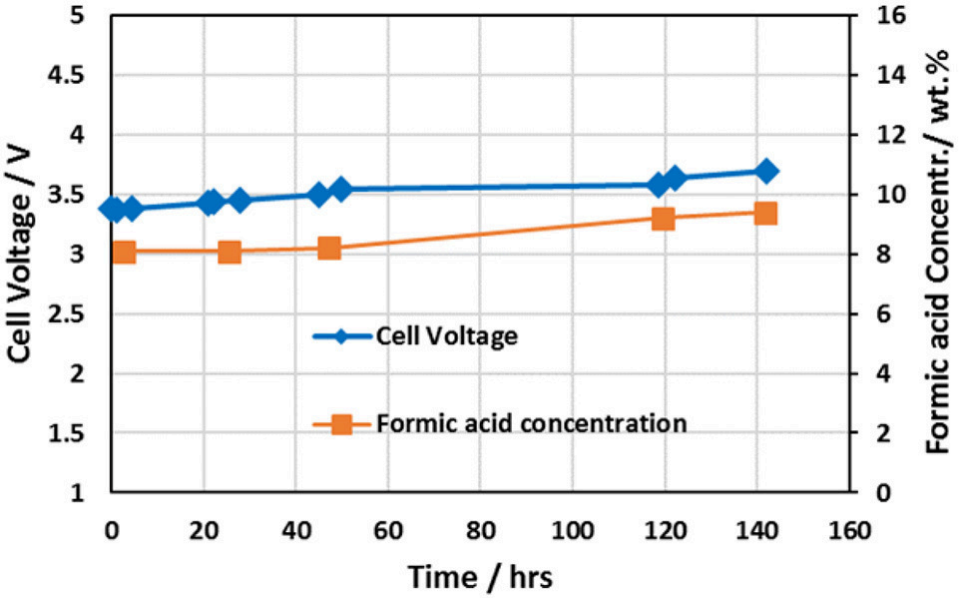
Formic acid cell performance data for the cell configuration tabulated in Table 2 utilizing an IrO_2_ catalyst coated titanium sintered fiber anode, Nafion® 324 cation membrane, and Sustanion® 37-50 anion exchange membrane.

Reference (Yang et al., 2017a) provides detailed performance information of the 3-compartment formic acid cell. Figure 4 shows the performance data for the 5 cm^2^ 3-compartment formic acid cell having a cell configuration utilizing an IrO_2_ coated titanium sintered fiber anode on a titanium flow field, Nafion® 324 cation membrane, and Sustanion® 37–50 anion exchange membrane operating at a current density of 140 mA/cm^2^. Table 2 provides the detailed cell configuration information. The cell was operated for about 142 h, producing a formic acid product concentration increasing from about 8.1 to 9.4% w%, and operating at a Faradaic efficiency of 80% at the start and at 94% near the end of the run until it was shutdown for inspection. No deterioration of the cell anode/cathode components was noted. The key in obtaining high FA Faradaic efficiency was employing a Nafion® 324 cation membrane, which minimized the transport of formate/formic acid ions into the anolyte compartment.

**Table 2:** Formic acid cell 142 h extended run electrolyzer configuration.

**Anode**
IrO_2_ based thermal coating on titanium sintered fiber anode on a titanium serpentine flow field, DI water anolyte
**Cathode**
GDE, Nanoparticle Sn, Sn Loading: 5 mg/cm^2^ 5 wt% PTFE, 5 wt% carbon black, 5wt % imidazolium-based Sustainion® 37-50 ionomer CO_2_ gas flow rate: 20 mL/min
**Anion Membrane**
Dioxide Materials Sustainion® X37-50 imidazolium-based anion exchange membrane
**Cation Membrane**
Chemours Nafion® N324
**Center Flow Compartment**
Resin Fill: Amberlite® IRC 120 Hydrogen form, 620-830 μm bead size DI water single-pass mode input flow rate: 0.10 mL/min
**Operating Temperature:** Ambient
**Operating Current:** 0.7 amps, CD: 140 mA/cm^2^
**Cell Active Membrane Area:** 5 cm^2^

The calculated formic acid cell design power consumption is in the range of 4.3–4.7 DC kWh/kg FA at these ambient operating conditions. This corresponds to an electrical operating cost of $214–$232 per ton of FA using a power cost of $0.05/kWh.

### Future scale-up and formic acid product applications

**Figure 5:**
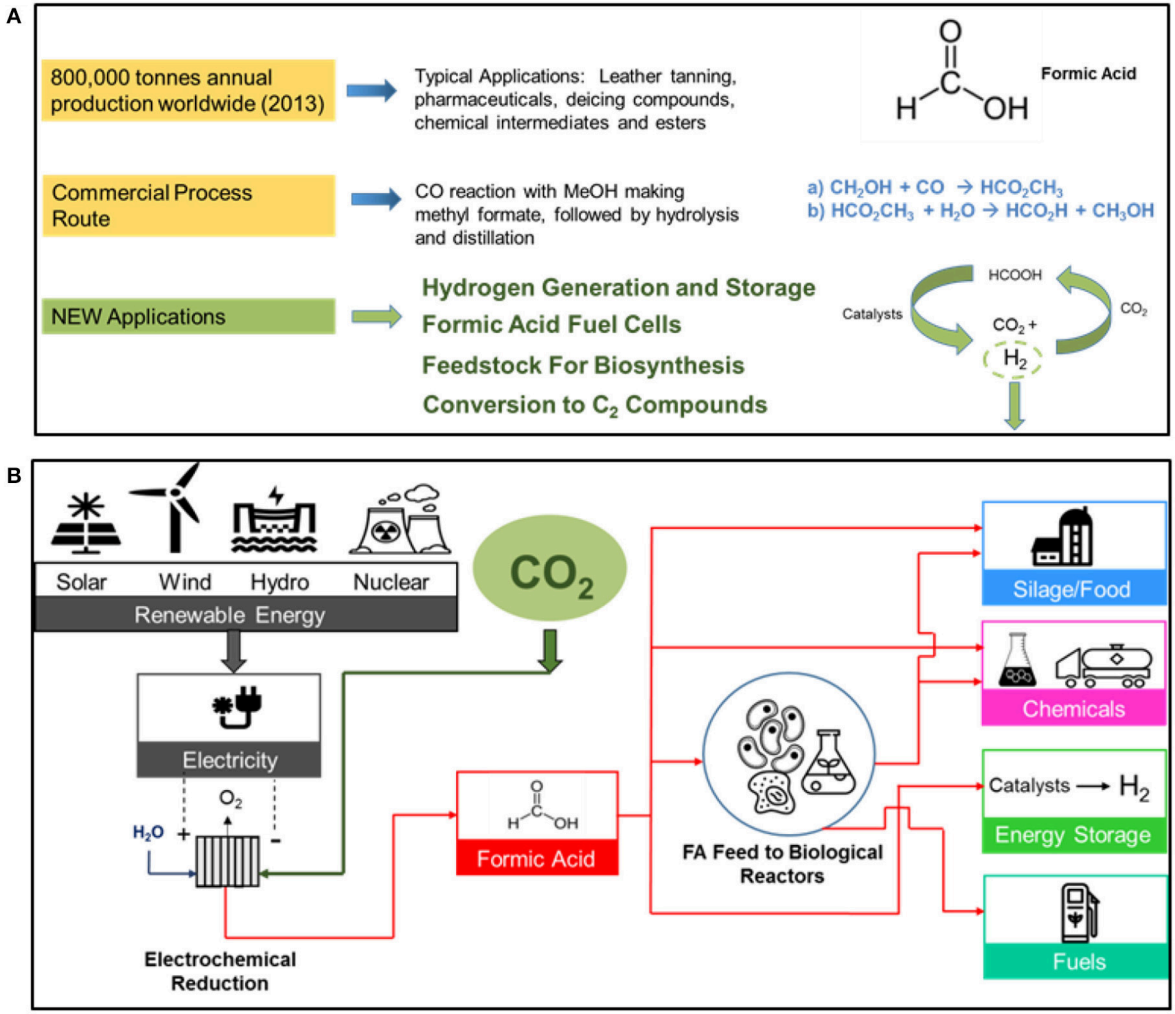
The worldwide market for formic acid (2013) showing the current chemical commercial process route by the carbonylation of methanol, and some new FA applications is shown in **(A)**. A sustainable route to formic acid using renewable energy resources and manufacturing products in the various product categories is shown in **(B)**.

The worldwide market for formic acid and the commercial chemical production route as well some product applications are shown in Figure 5A.

A sustainable route in producing formic acid from renewable energy resources is shown in Figure 5B. The various FA product applications include silage/food, chemicals, energy storage, and fuels. The use of formic acid as a feedstock in biological reactors, replacing sugars and/or agricultural grains, is another promising feedstock route.

DM is currently working with industrial partners in scaling up the electrochemical formic acid technology.

## Electrochemical CO_2_ electrolysis: conversion of CO_2_ to CO

Interest in the development of process methods of converting CO_2_ to a commercially useful and valuable product such as CO has been increasing over the last 20 years. CO in combination with hydrogen is the basis for the Fischer-Tropsch (F-T) process that is capable of producing a wide variety of fuels and chemicals. One important research area is using electrochemical processing in the conversion of CO_2_ to CO. Some of the research work in this area has been summarized in several review papers (Appel, 2013; Costentin et al., 2014; Masel et al., 2014a, 2016b).

The development of the DM-based CO_2_ electrolyzer technology in efficiently producing CO at high selectivities is based on three technological improvements. These are:

- Development of a GDE cathode structure comprising an imidazole-based ionomer as a co-catalyst with nanoparticle Ag, reducing the potential at the cathode for the CO_2_ reduction reaction to CO.
- Development of a high conductivity and alkaline stable anion membrane (Sustainion® membrane) that can conduct OH^−^ and bicarbonate/carbonate ions.
- Development of a GDE anode structure comprising a nanoparticle IrO_2_ catalyst that is stable for more than 3,000 h of operation.

All of these developments have helped create an electrochemical CO_2_ technology that can simultaneously operate at high current densities, high CO selectivity, and low cell potentials. Much of the recent work on the CO_2_ electrolysis technology are summarized in various papers and patents (Masel et al., 2014b, 2015; 2016c Masel and Rosen, 2014; Masel and Chen, 2015). Some of the aspects of the CO_2_ electrolyzer design, technology improvements, and performance are described in the next few sections.

### CO_2_ electrolysis cell design

**Figure 6:**
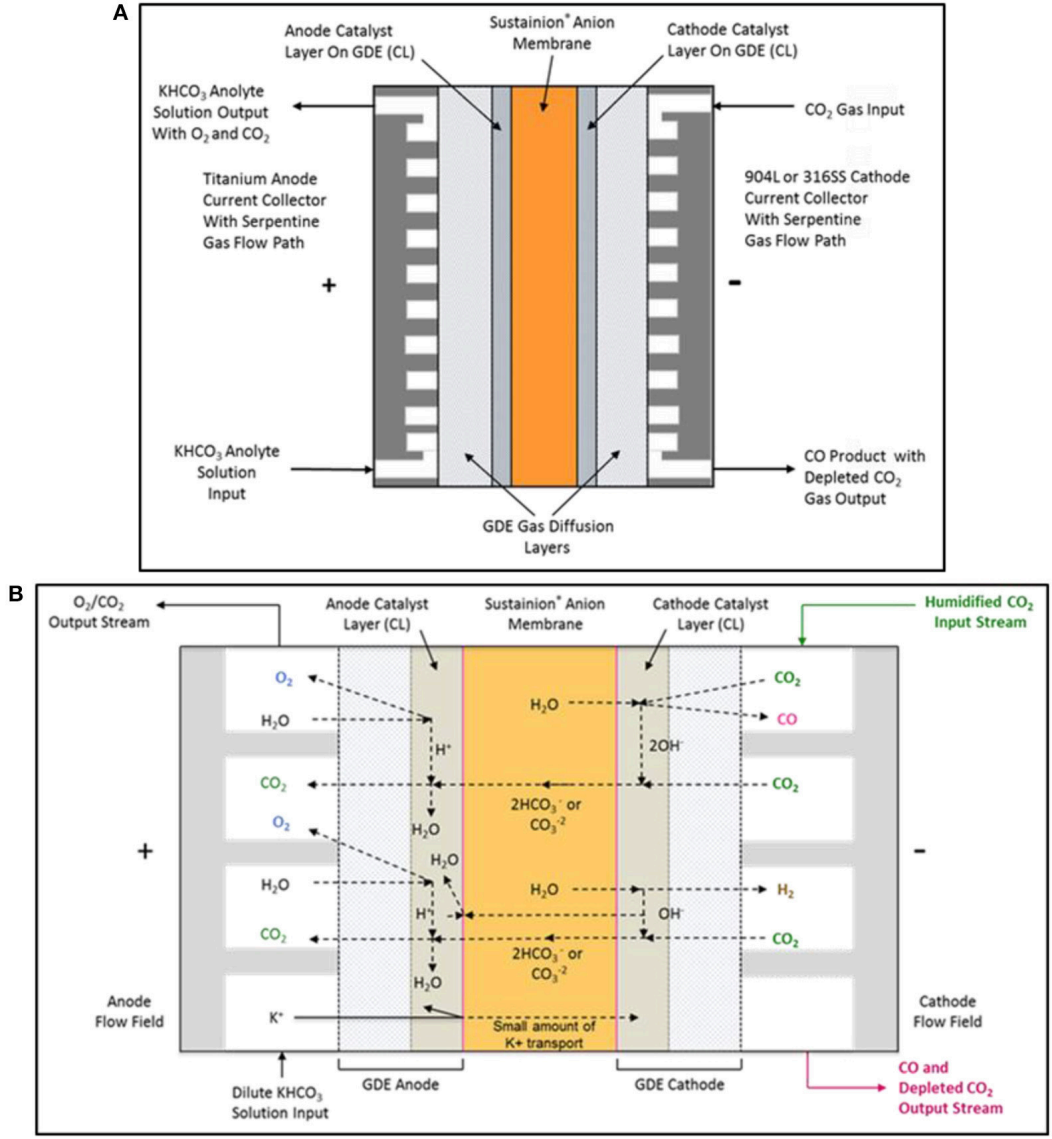
The DM electrochemical CO_2_ cell configuration is shown in **(A)**. The proposed electrochemical CO_2_ cell internal chemistry showing the main reactions and ionic specie flow paths are shown in **(B)**.

The basic DM CO_2_ electrolysis cell configuration is shown in Figure 6A. The cell design employs a titanium serpentine flow path anode current collector and a graphite or 316L stainless steel serpentine flow field cathode current collector. The GDE anode utilizes a nanoparticle IrO_2_ catalyst on a carbon paper support. The GDE cathode uses a nanoparticle Ag with Sustainion® imidazole-based ionomer catalyst combination on a carbon paper support that suppresses the formation of hydrogen at the cathode, thus obtaining very high selectivities of 95 to 99% of CO_2_ to CO.

### CO_2_ cell chemistry

Figure 6B shows the main complex ion flow paths in the electrochemical CO_2_ cell. The CO_2_ cell operates using a dilute 10 mM potassium bicarbonate solution anolyte and a humidified CO_2_ gas catholyte stream. High KHCO_3_ anolyte concentrations can result in the precipitation of the potassium salts in the cathode GDE, resulting in CO_2_ gas flow blockage in the cathode serpentine flow path.

#### CO_2_ cell cathode reactions

Below are the proposed cathode reactions in the CO_2_ cell. CO_2_ is reduced to CO on an Ag nanoparticle catalyst and formed hydroxide ions are transported through the anion exchange membrane or react with CO_2_ to form carbonate/bicarbonate anions which will also pass through the anion exchange membrane.

- CO_2_ reduction reaction on nanoparticle Ag catalyst producing CO and hydroxide ions:
  (5) $${\text{CO}}_{\text{2}}+{\text{H}}_{2}\text{O}+{\text{2e}}^{-}\to \text{CO}+{\text{2OH}}^{-}-0.52\text{V vs SHE }@\text{pH}7$$
- CO_2_ reaction with hydroxide ions forming carbonate anions:
  (10) $${\text{CO}}_{\text{2}}+{\text{2OH}}^{-}\to {\text{CO}}_{\text{3}}^{-2}+{\text{H}}_{2}\text{O}$$
- CO_2_ reaction with hydroxide ions forming bicarbonate anions:
  (11) $${\text{CO}}_{\text{2}}+{\text{OH}}^{-}\to {\text{HCO}}_{\text{3}}^{-}$$
- Carbonate ions further reaction with CO_2_ to form bicarbonate anions:
  (12) $${\text{CO}}_{\text{3}}^{-\text{2}}+{\text{CO}}_{\text{2}}+{\text{H}}_{2}\text{O}\to {\text{2HCO}}_{\text{3}}^{-}$$
- Water reduction electrolysis side reaction producing both hydrogen gas and hydroxide ions:
  (13) $${\text{2H}}_{\text{2}}\text{O}+\text{2e}-\to {\text{H}}_{2}+{\text{2OH}}^{-}$$

#### CO_2_ cell anode reactions

Below are the proposed anode reactions in the CO_2_ cell. The anode reactions are complex and may be a set of mixed reactions. Water may be electrolyzed on the nanoparticle IrO_2_ oxidation catalyst forming oxygen and hydrogen ions with an alternate possible anode reaction being the electrolysis of hydroxide ions at the catalyst, forming oxygen and water. The presence of the carbonate/bicarbonate ions in the anolyte and their conversion to CO_2_ provides complex bulk solution buffering in the anolyte compartment.

- Electrolysis of water on anode catalyst making oxygen and hydrogen ions at pH 7 and pH 0:
  (11) $${\text{2H}}_{\text{2}}\text{O}\to {\text{O}}_{2}+{\text{4H}}^{+}+{\text{4e}}^{-}\;0.815\text{V vs SHE }@\text{ pH 7}$$
  (13) $${\text{2H}}_{\text{2}}\text{O}\to {\text{O}}_{2}+{\text{4H}}^{+}+{\text{4e}}^{-}\;1.23\text{V vs SHE }@\text{ pH }0$$
- Electrolysis of hydroxide ions on the anode catalyst forming oxygen and electrons:
  (14) $${\text{4OH}}^{-}\to {\text{O}}_{2}+{\text{2H}}_{\text{2}}\text{O}+{\text{2e}}^{-}\;0.40\text{V vs SHE }@\text{ pH}$$
- Bicarbonate decomposition by hydrogen ions forming CO_2_ and water:
  (14) $${\text{HCO}}_{\text{3}}^{-}+{\text{H}}^{+}\to {\text{CO}}_{\text{2}}+{\text{H}}_{2}\text{O}$$
- Carbonate decomposition by hydrogen ions forming CO_2_ and water:
  (15) $${\text{CO}}_{\text{3}}^{-\text{2}}+{\text{2H}}^{+}\to {\text{CO}}_{\text{2}}+{\text{H}}_{2}\text{O}$$
- Hydroxide ions reacting with hydrogen ions producing water:
  (16) $${\text{H}}^{+}+{\text{OH}}^{-}\to {\text{H}}_{2}\text{O}$$

#### Overall CO_2_ cell reactions—determining if bicarbonate and carbonate are the predominant mobile ionic current carrier species

The complex ion transport in the CO_2_ cell is theorized to mainly consist of bicarbonate and carbonate anions as the predominant current carriers as well as any hydroxide anions that had not reacted with CO_2_ gas in the catholyte reactions at the GDE cathode. Experimental investigation of the ratios of the transport of these anion species in the cell and through the membrane have not yet been fully investigated. Testing of the DM 250 cm^2^ cell at LanzaTech (Skokie, IL) provided important data on the cell anolyte gas composition by GC. Table 3 shows some of the potential reactions based on (A) bicarbonate or (B) carbonate anion transport respectively in the cell. Net anode reaction (17) in Table 3A would predict that the anode gas composition should have a CO_2_ to O_2_ ratio of 4:1 or gas composition consisting of 80% CO_2_ and 20% O_2_ if bicarbonate was the only anion current carrier in the cell. Alternatively, net anode reaction (20) in Table 3B would predict a 2:1 ratio of CO_2_ to O_2_ or gas composition consisting of 67% CO_2_ and 33% O_2_ if carbonate was the only anion current carrier in the cell.

**Table 3:** Summary of reactions having **(A)** bicarbonate or **(B)** carbonate as the selected mobile anion specie through the anion membrane in the CO_2_ cell.

**A. BICARBONATE**		
Cathode:	CO_2_ + H_2_O + 2e^−^ → CO + 2OH^−^	(5)
	+ 2[CO_2_ + OH^−^ → ${\text{HCO}}_{3}^{-}$]	(7)
Net Cathode:	3CO_2_ + H_2_O + 2e^−^ → CO + 2${\text{HCO}}_{3}^{-}$	(16)
Anode:	2H_2_O → O_2_ + 4H^+^ + 4e^−^	(12)
	+ 4[${\text{HCO}}_{3}^{-}$+ H^+^ → CO_2_ + H_2_O]	(8)
Net Anode:	4${\text{HCO}}_{3}^{-}$ → 4CO_2_ + 2H_2_O + O_2_	(17)
Overall Net:	2CO_2_ → 2 CO + O_2_	(18)
**B. CARBONATE**		
Cathode:	CO_2_ + H_2_O + 2e^−^ → CO + 2OH^−^	(5)
	+ CO_2_ + 2OH^−^ → ${\text{CO}}_{3}^{-2}$ + H_2_O	(10)
Net Cathode:	2CO_2_ + 2e^−^ → CO + ${\text{CO}}_{3}^{-2}$	(19)
Anode:	2H_2_O → O_2_ + 4H^+^ + 4e^−^	(12)
	+ 2[${\text{CO}}_{3}^{-2}$ + 2H^+^ → CO_2_ + H_2_O]	(15)
Net Anode:	2${\text{CO}}_{3}^{-2}$ → 2CO_2_ + O_2_ + 4e^−^	(20)
Overall Net:	2CO_2_ → 2 CO + O_2_	(18)

DM's measurement of the cell anode gas composition in the past had showed variability in CO_2_ content, ranging from 30 to 60% depending on the operating conditions of the cell. One GC gas analysis set of an operating 250 cm^2^ CO_2_ cell at LanzaTech showed a gas composition as follows:

**Table:** 

Anolyte Gas Product:	64% CO_2_, 33% O_2_ by volume
Catholyte Gas Product:	39% CO, 57% CO_2_, 0.34% H_2_
CO Selectivity:	99.2%
Catholyte CO_2_ Gas Feed Rate:	1,000 cc/min
Operating Cell Current Density:	100 mA/cm^2^ (25 Amps)
Operating Cell Voltage:	2.89 V

The CO_2_ cell typically has a bulk anolyte pH of about 6.0 when operating on a 10 mM KHCO_3_ electrolyte, which is equivalent to a 50:50 mixture of carbonic acid (H_2_CO_3_) and bicarbonate. These anolyte gas results point to carbonate ions as potentially being the main charge carrier because the CO_2_:O_2_ molar gas ratio was about 2:1. So the definitive answer to the predominant ionic charge carriers in the CO_2_ cell will need to be further investigated.

### CO_2_ cell performance

Conducting long term tests of any electrochemical cells is the key in determining the performance and stability of all the cell components, particularly membranes and the anode/cathode catalysts. Here we discuss some of the long term performance of the DM laboratory 5 and 250 cm^2^ CO_2_ electrolyzer cells.

#### 5.0 cm^2^ CO_2_ cell long term performance

**Figure 7:**
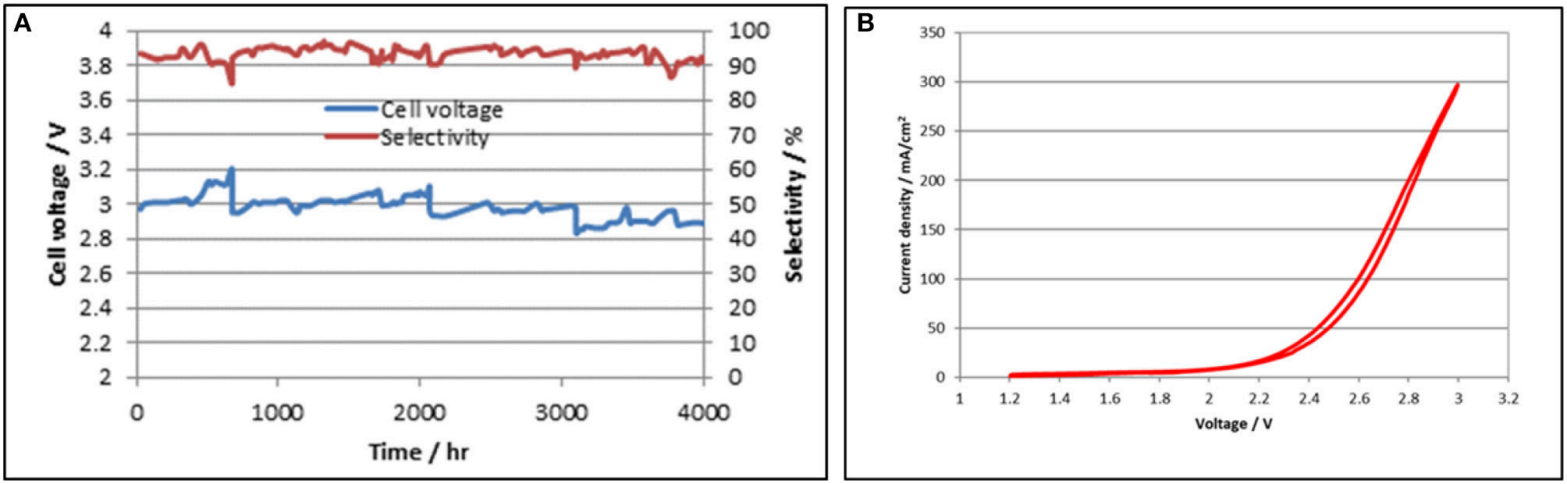
5.0 cm^2^ long term cell test data showing stable performance for 4,000 h is shown on the left in **(A)**. A cell voltage polarization plot for the 5.0 cm^2^ CO_2_ cell operating at room temperature is shown on the right in **(B)**.

The DM CO_2_ cell testing program employs numerous cells, evaluating different Sustainion® anion membrane compositions as well as anode and cathode composition configurations for operational periods of up to 4,000 h and more to ensure that the desired cell membrane and catalyst configuration performance is stable. Cell operation details and data have been detailed in several papers (Rosen et al., 2013; Liu et al, 2015; Masel and Chen, 2015). An example of a 5.0 cm^2^ cell long term test run for 4,000 h is shown in Figure 7A. A cell voltage polarization plot for the 5 cm^2^ CO_2_ cell operating at room temperature (24°C) is shown in Figure 7B.

#### 250 cm^2^ design CO_2_ cell performance

**Figure 8:**
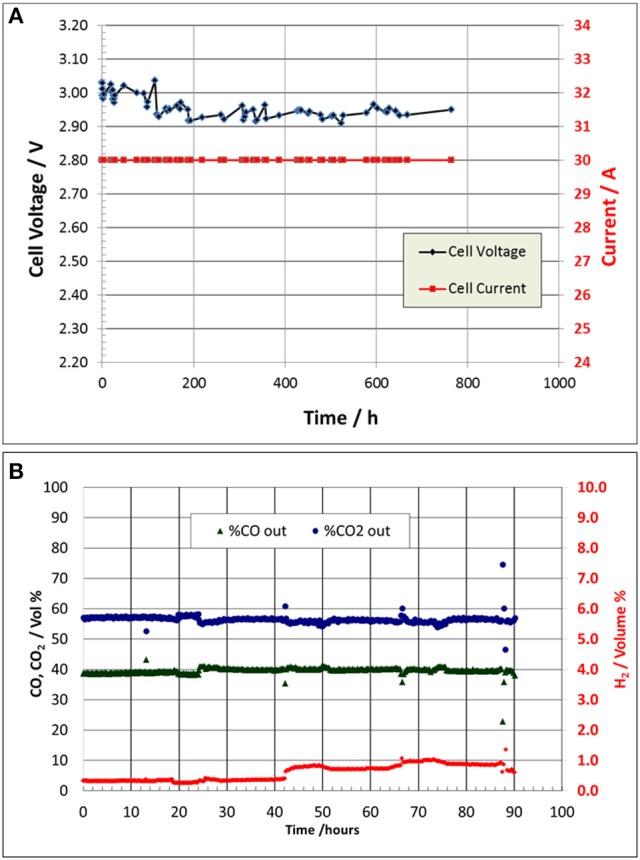
The 250 cm^2^ cell performance at DM showing stable voltage operation for about 760 h until shutdown is shown in **(A)**. The 250 cm^2^ CO_2_ operation at LanzaTech shows the cell catholyte output GC gas composition analysis in an extended performance 90 h test run is shown in **(B)**.

The 5.0 cm^2^ CO_2_ cell was subsequently scaled-up to a cell having a geometric active area of 250 cm^2^. In addition to testing at DM, one cell was sent to LanzaTech (Skokie, IL) for comparative testing. Table 4 shows the cell configuration details. The cell at DM was operated for a total of 760 h until shut down in preparation for further test work at LanzaTech. Figure 8A shows the cell operating performance at DM showing a stable voltage range of 2.9–3.0 V over the test period. The cell operating current density was 120 mA/cm^2^, with an anolyte operating temperature of about 45°C, without the need for any external cooling.

**Table 4:** 250 cm^2^ CO_2_ cell configuration details.

**250 cm^2^ cell construction details:**
Fuel Cell Technologies, Model 250SCH, modified with titanium anode and graphite flow fields DM Sustainion® T ePTFE reinforced membrane (88 μm wet thickness) Anode GDE: IrO_2_ on 5% CFP (carbon fiber paper) Cathode GDE: Nanoparticle Ag/Sustainion® ionomer on Sigracet 39BC paper
**Operation:**
Anolyte solution flow: 200 mL /min, 10 mM KHCO_3_ CO_2_ gas rate: 1,000 mL/min, hydrated with water vapor

#### 250 cm^2^ CO_2_ cell test operation at LanzaTech

The 250 cm^2^ CO_2_ cell operation at LanzaTech helped in confirming the CO_2_ cell CO selectivity performance and gas product compositions. Figure 8B shows the test results of the 90 h test run. LanzaTech employed an automated Agilent Technologies Model 490 Micro GC with a Mol Sieve 5A (MS5A) column to analyze and monitor the cell catholyte gas product composition automatically over time. The outlier points are occasional analysis blips in the GC analysis. The cell CO selectivity ranged from 97.5% (i.e., 1% H_2_ content) to about 99% (0.38% H_2_ content).

#### 250 cm^2^ CO_2_ cell operating system

**Figure 9:**
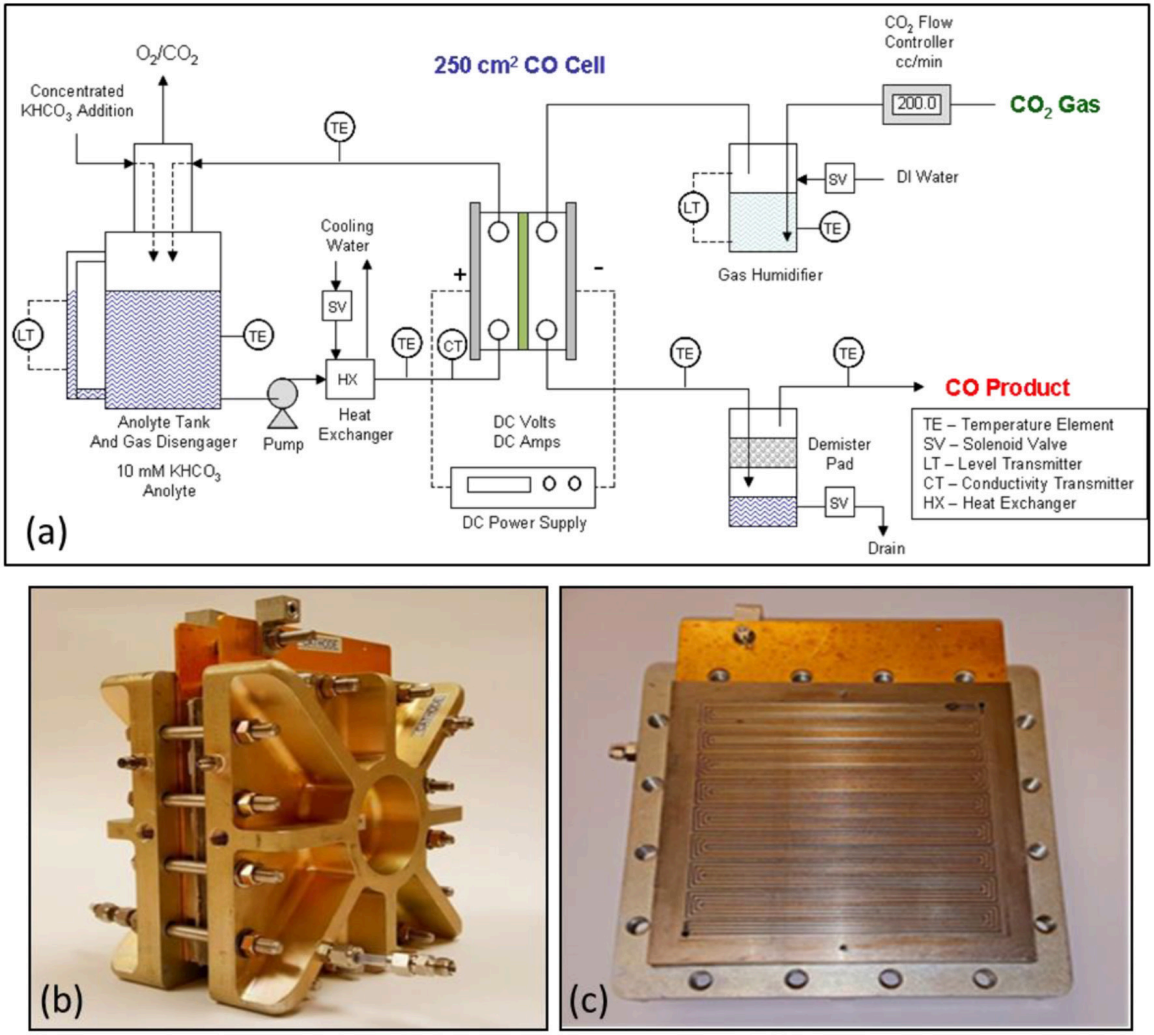
Schematic of the 250 cm^2^ CO_2_ cell experimental test system is shown in **(a)**. The DM modified 250 cm^2^ CO_2_ electrolyzer cell as shown in **(b)** on the left. The design used a titanium serpentine anode flow field design as shown in **(c)**.

Figure 9a shows the 250 cm^2^ CO_2_ cell experimental test system configuration. Figures 9b,c show the Fuel Cell Technologies cell that was modified for use in the DM 250 cm^2^ CO_2_ cell design. The left photo Figure 9b shows the entire cell and right photo Figure 9c shows the titanium serpentine anode flow field design that was fabricated for the cell. CO_2_ gas was metered using a CO_2_ gas flow controller and bubbled through a water filled gas humidifier tank. The humidified CO_2_ gas enters the top of the CO_2_ cell cathode and exits at the bottom as a depleted CO_2_ gas stream containing the CO product and a small amount of byproduct H_2_. Excess condensed water is collected and separated from the gas stream product. The typical gas product from the CO_2_ cell was mainly CO with a small amount of byproduct H_2_ and operating with a stoichiometric excess of CO_2_. The cell CO selectivity ranged from about 95 to 99% depending on the applied current density and amount of excess CO_2_ over stoichiometric used in the catholyte gas feed as shown in Figure 8. The anolyte loop consists of a 10 mM KHCO_3_ electrolyte, having a conductivity of about 1.0 mS, which was pumped through the cell GDE anode at selected flowrates. A heat exchanger was used to control the anolyte loop temperature by controlling the flow of cooling water. A conductivity controller was used to control the addition of a concentrated KHCO_3_ solution to the anolyte loop as needed to maintain anolyte conductivity.

### CO_2_ cell scale-up progress

Work on scale-up of the DM CO_2_ cell is continuing with other partners. In addition to scale-up, development work is proceeding in finding suitable stable non-precious metal catalyst replacements for the nanoparticle IrO_2_ used in the anode GDE design.

An estimate of the CO_2_ cell process power consumption, using the 250 cm^2^ cell data of 2.95 V at 30 amps (120 mA/cm^2^) with 98% CO selectivity, was calculated to be 5.8 DC kWh/kg of CO at 45°C.

## Alkaline water electrolysis

Alkaline water electrolysis is recognized as a mature technology that is reliable and safe, with electrolyzers having operational lifetimes as long as 15 years. Their biggest advantage is that they employ abundant, non-precious metal electrode catalysts. The conventional alkaline water electrolyzer cell design typically uses a diaphragm type separator. The separator typically requires that the distance between the anode and cathode electrodes to be about 2–3 mm apart to prevent gas crossover, thus limiting the operating current density to about 200 mA/cm^2^ in order to operate at a reasonable operating voltage and corresponding overall energy efficiency. In order to increase the current density, while maintaining the same or higher energy efficiency, the next technology step would be to develop stable alkaline stable anion exchange membranes that would allow true zero-gap water electrolyzers. DM has developed alkaline water electrolyzer technology based on the development of a stable anion exchange membrane. The development work is summarized in various published papers and patents (Pletcher and Li, 2011; Deavin et al., 2012; Appel, 2013; Rosen et al., 2013; Masel et al., 2016b; Kutz et al., 2017; Liu et al., 2017a,b).

### DM alkaline water electrolyzer cell design

The development of an alkaline stable Sustainion® anion exchange membrane has enabled the development of zero-gap design CO_2_ electrolyzers that can actually improve current density by an order of magnitude at the same or lower cell voltage as compared to the current diaphragm-based alkaline water electrolyzers. In order to test Sustainion® anion exchange membrane in a water electrolyzer configuration, a decision was made to use a similar cell design, Figures 9A,B, as employed in the CO_2_ electrolyzer, but using 316L stainless, or more preferred, pure nickel 200 flow fields in both the cathode and anode designs.

### Alkaline electrolysis cell chemistry

The alkaline water electrolysis cell operates using a 1 M KOH electrolyte solution at temperatures from ambient to 60°C. Operation at 60°C provides the lowest operating cell voltage using the current Sustainion® anion exchange membranes to separate the anode and cathode reactions.

#### Alkaline electrolysis cell electrode reactions

Sustainion® membranes, which are prepared in the chloride (Cl^−^) form, need to be converted to the hydroxide (OH^−^) form. This is done by soaking in 1 M KOH for at least 12 h. In the presence of a KOH electrolyte, both cathode and anode are operating at a local pH is close to 14. At the cathode, the hydrogen evolution reaction (HER) uses water as proton donor, producing hydrogen and OH^−^ (Equation 6). OH^−^ ions transport from cathode to anode and recombine at the anode to generate O_2_ and electrons (Equation 14). The overall reaction is written in Equation 21.

(6) $${\text{4H}}_{\text{2}}\text{O}+{\text{4e}}^{-}\to {\text{2H}}_{\text{2}}+{\text{4OH}}^{-}\;{\text{E}}_{\text{c}}^{0}=\text{0}.\text{826 V vs SHE}$$

(14) $${\text{4OH}}^{-}\to {\text{O}}_{2}+{\text{2H}}_{\text{2}}\text{O}+{\text{4e}}^{-}\;{\text{E}}_{\text{a}}^{0}=-0.404\text{ V vs SHE}$$

(21) $${\text{2H}}_{\text{2}}\text{O}\to {\text{2H}}_{\text{2}}+{\text{O}}_{2}\;{\text{E}}^{0}=1.23\text{ V}$$

## Laboratory alkaline water electrolysis test system

**Figure 10:**
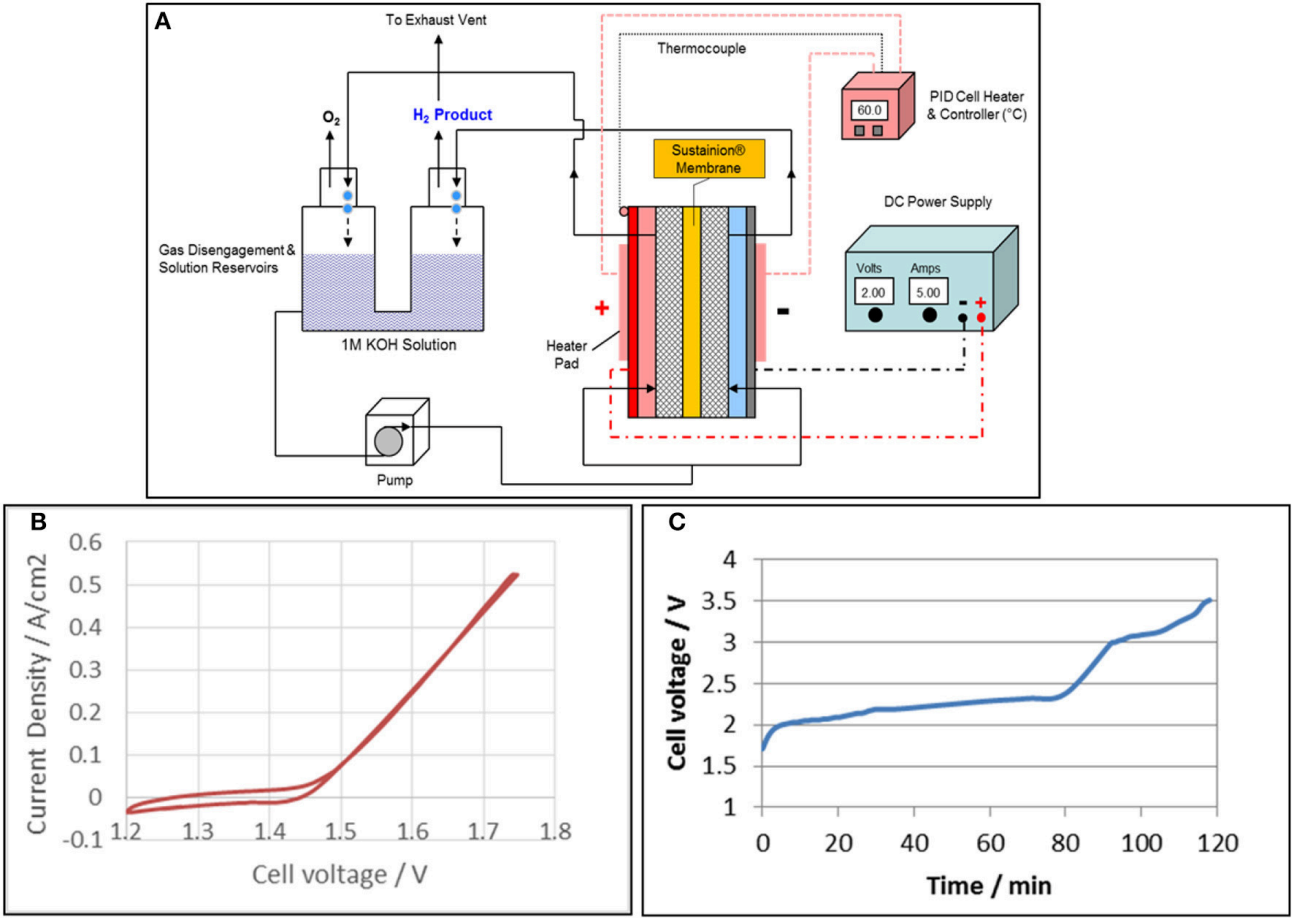
Alkaline water electrolysis experimental test system for 5 cm^2^ test cells is shown in **(A)**. A typical cyclic voltammogram of the alkaline water electrolyzer with a Sustainion® anion exchange membrane based on functionalization with 1-methyl imidazole is shown in **(B)** and the corresponding cyclic voltammogram is shown in **(C)**. A Pt cathode and IrO_2_ anode were used and the cell was operated in 1M KOH at 60°C.

Figure 10A shows a general diagram of a DM 5 cm^2^ experimental alkaline water electrolysis operating system. The bottom of electrochemical cell anolyte and catholyte compartments are separately fed a pumped stream of 1 M KOH from a common gas disengagement 1 M KOH solution reservoir. Both the anolyte and catholyte compartment solution/gas product streams are separately routed to the individual O_2_ and H_2_ gas disengager sections of the gas/liquid disengager unit. The O_2_ and H_2_ gas products are separately vented from the disengager. One DM cell design uses silicone flexible strip heater pads mounted on the anode and cathode external plates to supply the heat for cell operation at 60°C. The cell temperature is controlled using a PID temperature controller using a thermocouple mounted on the cell anode or cathode body.

### Alkaline water electrolysis cell performance

Figure 10B shows typical cyclic voltammogram of the alkaline water electrolyzer with a Sustainion® anion exchange membrane based on functionalization with 1-methyl imidazole. Pt and IrO_2_ black were used as cathode and anode, respectively. The onset potential for water electrolysis with anion exchange membrane started at 1.42 V, and the current increased quickly at a current density of 0.52 A/cm^2^ (the maximum current of potentiostat) was 1.74 V. The long term test was conducted at constant current of 0.8 A/cm^2^ using a power supply, and the results are shown in Figure 10C. The cell voltage reached 2 V in 5 min, and gradually increased to 2.3 V in 80 min. The cell voltage then increased quickly and reached a cut off voltage of 3.5 V in less than 120 min. These results showed that the 1-methyl imidazole-based membrane works in an alkaline water electrolyzer, but was not stable in 1 M KOH. This was due to the three active hydrogen molecules in the imidazole ring that are attacked by hydroxyl radicals (·OH) in 1 M KOH, especially at higher temperatures.

In order to improve the stability of anion exchange membrane, tetramethyl imidazole was grafted onto the styrene backbone instead of 1-methyl imidazole. This membrane was named Sustainion® 37–50. Figure 10C shows the polarization curves of the cell with Sustainion® 37–50 membrane running in 1 M KOH at 60°C. The cell with Pt cathode and IrO_2_ anode achieved current densities of 1 and 3 A/cm^2^ at 1.62 and 1.77 V, respectively. As a comparison, the cell with NiFeCo cathode and NiFeOx anode reached 1 and 3 A/cm^2^ at 1.90 and 2.13 V, respectively. The cell with base metal/metal oxide electrodes and Sustainion® 37–50 anion exchange membranes achieved a 5-fold higher current density at 1.90 V than that (200 mA/cm^2^ at 2.0 V) of the commercial alkaline water electrolysis with diaphragms as separator.

**Figure 11:**
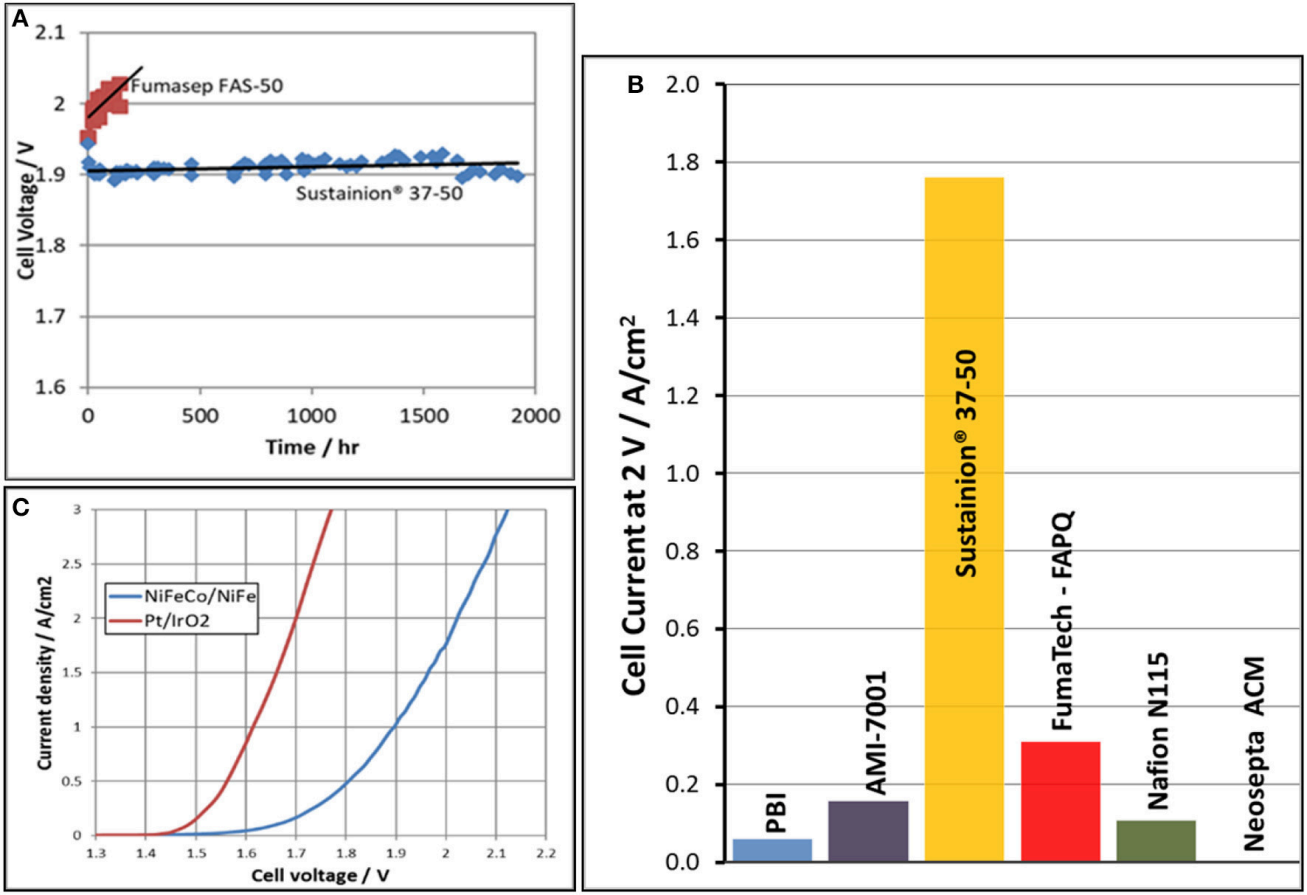
Cell voltage as function of time comparison in cells evaluating a Sustainion® 37-50 anion membrane and a Fumatech Fumasep® FAS-50 anion exchange membrane in 1M KOH at 60°C is shown in **(A)**. The alkaline electrolysis cell current at an applied voltage of 2 V for various membranes in 1M KOH at 60°C is shown in **(B)**. The polarization curves of the same cell comparing precious metal (Pt/IrO_2_) electrode catalysts to base metal electrode catalysts (NiFeCo/NiFe) in 1M KOH at 60°C using a Sustainion® 37-50 anion exchange membrane is shown in **(C)**.

Two questions were then needed to be answered. Does the Sustainion® anion exchange membrane have long term KOH stability and how long can it maintain performance? Figure 11A compares the cell performance of the Sustainion®37–50 anion exchange membrane with a commercial Fumatech FAS-50 anion exchange membrane in identical DM water electrolysis cells operating at a constant current density of 1 A/cm^2^ in 1 M KOH at 60°C. The results show that the Sustanion® membrane cell, operating at 1 A/cm^2^ for almost 2,000 h, had a cell voltage increase rate of only 5 μV/h. This indicated that Sustainion® 37–50 anion exchange membrane functionalized with 1,2,4,5-tetramethylimidazole was chemically very stable in KOH compared to the membrane functionalized with 1-methyl imidazole. This also confirmed that the instability of the 1-methyl imidazole functionalized membrane was due to the active hydrogen in the ring. The Fumasep FAS-50® anion exchange membrane showed a significant increase of 200 μV/h in cell voltage over time and exhibited a high cell leakage current of 0.2 A/cm^2^ at the 200 h point, and was then shut down.

Figure 11B shows alkaline electrolysis cell current at an applied potential of 2 V in evaluating the performance of various commercial membranes using a NiFeCo catalyst coated cathode and NiFeOx catalyst coated anode operating in 1 M KOH at 60°C. The Sustainion® anion exchange membrane showed the highest cell current at the applied voltage. This compares with the membrane ASR membrane measurement data shown in Table 1.

Figure 11C shows the polarization curves of the same cell comparing precious metal electrode catalysts (Pt on cathode/IrO_2_ on anode) to base metal electrode catalysts (NiFe on anode/NiFeCo on cathode) using a Sustainion® 37–50 anion exchange membrane in 1 M KOH at 60°C. At a current density of 1 A/cm^2^, the cell using the precious metal catalysts operated at a cell voltage of 1.63 V, and the base metal catalysts cell operated at 1.9 V. The alkaline electrolysis cell CAPEX (capital expenditure) and OPEX (operating cost) which will need to include the availability/cost of both Ir and Pt catalysts in worldwide large scale installations then become important variables in the analysis decision for installing these systems.

## Alkaline water electrolysis cell scale-up

Commercial alkaline water electrolysis operate cells with electrode geometric areas varying from several hundred cm^2^ up to several m^2^. DM is now scaling up the zero-gap alkaline water electrolyzer design from 5 cm^2^ to larger 100–600 cm^2^ cells. In operating these larger alkaline water electrolyzers at high current densities of 1 A/cm^2^ or more, both water and heat management are critically important. Based on energy and mass calculations, operating a 100 cm^2^ cell at 1 A/cm^2^ and 1.90 V, the water consumption would be about 806 mL per day with the generation of about 67 W of waste heat that has to be removed. The waste heat for a single cell can be handled with air cooling, but a large cell stack would require a recirculating cooling water system having a heat exchanger. A deionized water make-up system would be required for maintain the system liquid KOH electrolyte concentration and volume.

The power consumption estimate for the alkaline water electrolysis cell, operating at 1 A/cm^2^ at 1.90 V, was calculated to be 50.5 DC kWh/kg H_2_. The electrolyzer energy consumption will be lower at lower operating current densities, but the capital costs for the electrolyzer cells will be significantly higher. All of these factors have to be considered when comparing the electrolyzer H_2_ power consumption to the DOE electrochemical hydrogen target goals which have stack energy efficiencies of 43 kWh/kg and hydrogen levelized cost of $2.00 for 2020 (U.S. Department of Energy (DOE), 2018).

## Summary

Electrochemical technology in the conversion of CO_2_ to various products such as formic acid and CO as well as hydrogen using alkaline water electrolysis cells is advancing to commercialization with the development of the new Dioxide Materials Sustainion® anion exchange membranes. These membranes are both highly conductive and show excellent stability in highly alkaline solution environments at temperatures up to 60°C. The electrochemical systems utilizing these new membranes are showing good stability in long term bench-scale tests over thousands of hours of operation. These anion exchange membranes are still in the development stage, and work is being done in improving the membrane mechanical and ionic conductivity properties.

Future work will focus on improvements in these anion exchange membranes as well as in the development of improved catalysts that will extend the performance of all these electrochemical technologies.

## Author contributions

JK, HY, ZL, SS, and RM have all fully contributed to the content in this article. JK, HY, and SS worked in the development of the formic acid technology. ZL, SS, RM, and JK all worked on the alkaline water electrolysis technology. SS, ZL, and RM have all worked on the development of the Sustainion® anion exchange membrane development. ZL, HY, and RM have all worked on the development of the CO_2_ electrolysis technology. All have contributed to the content and editing of this article.

### Conflict of interest statement

The authors have submitted several patents on the membranes and designs disclosed here including US 9,370,773, US 9,481,939, US 9,580,824, and US patent applications 15/400,775, 15/406,909, 15/411,831. The authors are all employees of Dioxide Materials and have a financial interest in these patents. Dioxide Materials is offering all of our research materials (membranes, catalysts etc.) for sale to other research groups so that they can reproduce and build on the findings.
